# HSP90 mediates the connection of multiple programmed cell death in diseases

**DOI:** 10.1038/s41419-022-05373-9

**Published:** 2022-11-05

**Authors:** Caiwang Peng, Fengyan Zhao, Hengli Li, Ling Li, Yantao Yang, Fang Liu

**Affiliations:** 1 College of Pharmacy, Hunan University of Chinese Medicine, Changsha, China; 2 Hunan Engineering Technology Center of Standardization and Function of Chinese Herbal Decoction Piece, Changsha, China; 3 Key Laboratory of Modern Research of TCM, Education Department of Hunan Province, Changsha, 410208 China

**Keywords:** Cell death, Diseases

## Abstract

Heat shock protein (HSP) 90, an important component of the molecular chaperone network, is closely concerned with cellular signaling pathways and stress response by participating in the process of maturation and activation of client proteins, playing a crucial role both in the normal and abnormal operation of the organism. In functionally defective tissues, programmed cell death (PCD) is one of the regulable fundamental mechanisms mediated by HSP90, including apoptosis, autophagy, necroptosis, ferroptosis, and others. Here, we show the complex relationship between HSP90 and different types of PCD in various diseases, and discuss the possibility of HSP90 as the common regulatory nodal in multiple PCD, which would provide a new perspective for the therapeutic approaches in disease.

## Facts

1. HSP90 takes part in multiple PCD by regulating the stability and function of clients.
2. HSP90 inhibitors are widely used in various diseases by affecting multiple PCD.
3. HSP90 involves in the crosstalk of multiple PCD.

## Introduction

PCD is commonly found that relates to embryonic development, immune response, aging and other physiological processes, playing an important role in cellular homeostasis by removing damaged and senescent cells [1]. Besides the well-known apoptosis, some non-apoptotic PCD forms such as ferroptosis and necroptosis are gradually discovered, which have been demonstrated that may occur together to maintain a normal cell cycle for avoiding internal and external stimuli [2–4]. Therefore, it may bring a new trend for the therapy of diseases based on the special mechanism clarification. However, the complex crosstalk of multiple PCD processes are not independent of each other but sharing a coordinated system to mediate pathology, resulting in the difficulty to affect them as expected synchronously. For example, features of necroptosis and ferroptosis can be observed in the model of acute kidney injury which could be alleviated by necroptosis inhibitor Nec-1 and ferroptosis inhibitor Fer-1, though the inhibition of ferroptosis is beneficial to necroptosis [5]. The deep studies of relevant regulatory mechanisms focusing on the crosstalk and interaction among various PCD, caspase family proteins, HSPs and other proteins that acted as common regulatory nodal have attracted widespread attention [6, 7].

Among those, HSPs, as a molecular chaperones, are synthesized to maintain homeostasis when cells are under diverse physiological states, which are considered to involve in various pathways like hormone and cell cycle [8], including constituent proteins (HSP40 and HSP90), and inducible proteins (HSP70 and HSP27) [9]. The different classes of HSPs exert specific function, and also could work together to maintaining proteostasis. As the typical representative HSPs, HSP90 and HSP70 have received the most attention belongs to the important part of the chaperone network [10]. Therefore, their roles in the pathogenesis and treatment of diseases are revealed gradually, including the regulation of proteostasis, immune and cell death pathways [11]. And the HSP90 appears to be a critical regulator of PCD.

HSP90 is the most abundant class in HSPs, whose expression could reach up to 4%-6% under stress conditions, and about 600 clients have been found in mammal [12, 13]. Generally, HSP90 fulfills the chaperone function by forming complexes with the co-chaperone and client to maintain the stability, processing and function of organisms [14]. For instance, binding to HSP90 is an indispensable part of the activation of some kinases and steroid receptors [15, 16]. As a wide range of clients, HSP90 is related to the development of lots of diseases by mediating key proteins of PCD, including receptor-interacting serine/threonine kinase (RIP) 1 in necroptosis, glutathione peroxidase (GPX) 4 in ferroptosis, Beclin-1 in apoptosis and autophagy [17–19].

The HSP90/co-chaperone/client complex is considered to be an emerging target for the treatment of diseases. So, how to block the interactions of HSP90 and client is gradually investigated, and inhibitors were proved to be the most commonly effective strategy [20–23]. Relevant genomics and experimental studies demonstrated that HSP90 inhibitors are potential antitumor agents, and some of them like geldanamycin (GA) and 17-allylamino-17-demethoxy-geldanamycin (17-AAG) have been developed and advanced into clinical trials [24, 25]. In consideration of the importance of PCD and the relation of HSP90 in the disease treatment, accumulating evidence indicates that HSP90 connects to multi-PCD such as apoptosis, necroptosis and ferroptosis [18, 26]. So, we summarized the latest researches on the correlation between HSP90 and PCD in diseases.

## HSP90

### HSP90 and co-chaperone

**Fig. 1:**
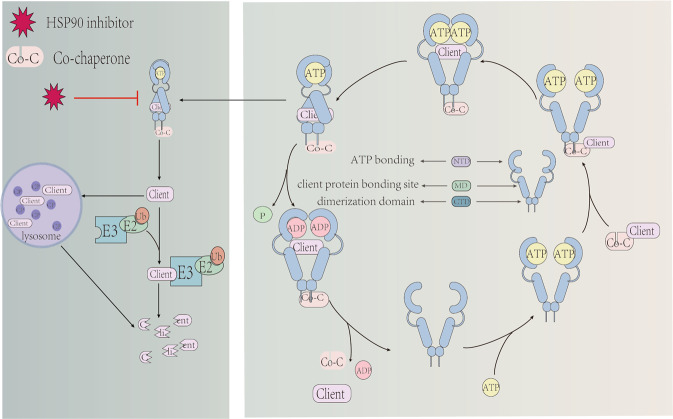
HSP90 regulates the stability and degradation of clients. Client is regulated by forming the complex of HSP90, co-chaperone and client, whose prerequisite is the ATP-binding HSP90. When the complex complete assembly, the conformation of HSP90 changes and ATPase is activated which takes participate in the process of client. The inhibition of HSP90 affects the complex to promoting client unstable, leading the degradation of client by lysosome and ubiquitinated protease pathways.

HSP90 is highly conserved during evolution, containing three conservative domains of C-terminal domain(CTD), middle domain(M-domain) and N-terminal domain(NTD) [13]. There is an ATP-binding site in NTD which is responsible for mediating the combination of co-chaperone and client based on ATPase activity [16]. The M-domain provides a site for ATP hydrolysis as well as binds to client [14], and CTD is related to the dimerization [27]. After dimerization of HSP90 connecting to ATP, the client binds to the M-domain, accompanied by NTD from open into closure [28] (Fig. 1). In general, there are four isoforms of HSP90 in cells which involves in various cellular functions, including HSP90α and HSP90β in the cytoplasm, tumor necrosis factor receptor-associated protein-1 (TRAP1) in the mitochondria and the glucose regulated protein 94 in the endoplasmic reticulum [8]. As an essential carrier of HSP90, the combination of client and HSP90 is decided by the modification of HSP90 and co-chaperone. It was reported that the moderate phosphorylation of HSP90 contributes to the maturation of most clients, but the hyperphosphorylation shows a negative effect on the chaperone machine [29]. In addition, the acetylation or the S-nitrosylation of HSP90 also blocks the interaction between HSP90 and client, which results in the inactivation and degradation of the client [12, 30].

Co-chaperone is also related to client activity, which assists with the function of HSP90 and provides selectivity for HSP90 by regulating its ATPase cycle, including p23, cell division cycle (CDC37), and so on [12, 31]. For instance, p23 is beneficial to stabilize the conformation of steroid receptors, but CDC37 is mainly responsible for kinase [32, 33]. Similarly, the inhibitors are also affecting clients selectively. The HSP90 inhibitor gedunin induces the degradation of steroid receptors in a dose-dependent manner, but has little effect on the stability of the kinase client [34]. Another inhibitor FW-04-086 regulates the cancer-related kinase to reduce proliferation and inducing apoptosis in breast cancer cells [35, 36]. Overall, the assembly of the HSP90/co-chaperone/client complex is closely relevant to various diseases, which promotes the application of HSP90 inhibitors.

HSP90 inhibitors are mainly used in cancer research, divided into NTD inhibitors, M-domain inhibitors and CTD inhibitors according to the action sites [37]. Some NTD inhibitors like GA impact the ATP binding pocket of HSP90, to promote the degradation of clients through lysosomal and ubiquitinated protease pathways [38, 39]. However, the compensatory expression of HSP70 would be induced by NTD inhibitors, which acts as a cytoprotection [40]. On the contrary, inhibitors designed for interacting with M-domain or CTD will not induce HSP70-dependent cell survival [33]. An M-domain inhibitor KA blocks the interaction between HSP90 and CDC37 by combining Cys420 with HSP90 while reserving its ATPase activity [41]. Except for the interaction with three domains of HSP90, inhibitors can also impact the co-chaperone directly. As seen in the study of Patwardhan et al., gedunin induces the cleavage of p23 by recruiting caspase7 in NTD, which decreases the interaction between HSP90 and p23 and affects the client subsequently [34, 42].

### HSP90 and diseases

**Fig. 2:**
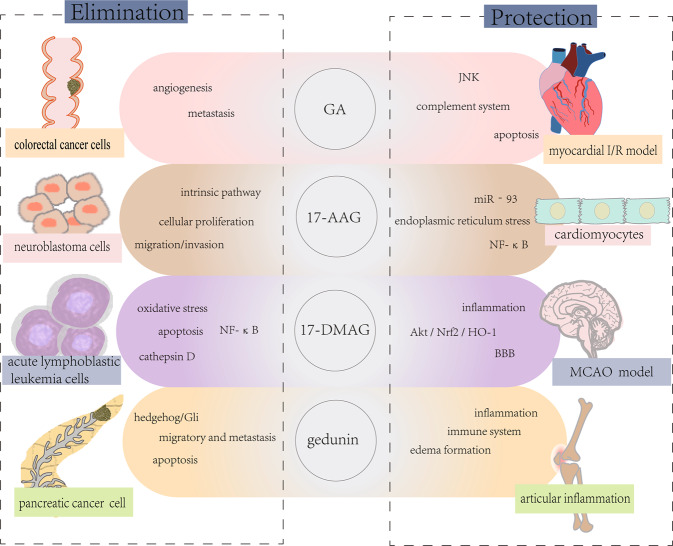
HSP90 inhibitors exert therapeutic effects in various diseases. HSP90 inhibitors could simultaneously apply to cancer, myocardial ischemia and other diseases. On the one hand, HSP90 inhibitors protect cardiomyocytes and neurons by reducing inflammation and relevant signaling pathways. On the other hand, inhibitors eliminate cancer cells by inducing apoptosis.

The enhancive synthesis and release of HSPs are beneficial to maintain cellular homeostasis [43, 44]. Generally, highly expressed HSP90 shows a positive cytoprotection and the cell viability is decreased by the inhibition or knockdown of HSP90, which is considered to be a feasible therapeutic target in cancer such as colorectal cancer, lung cancer and gliomas [21, 26, 45]. The main mechanism HSP90 inhibitors acted on cancer cells is mediated by inflammation and PCD, and similar events also have been found in ischemic diseases, neurodegenerative diseases, and others [7, 8, 46] (Fig. 2). For example, apoptosis could be induced in ovarian carcinoma cells when HSP90 is knockout [47]. But in the model of cerebral ischemia, treating with GA is conductive to the inhibition of apoptosis. And further more relevant information is listed in the table below [48] (Table 1).

**Table 1:** Inhibition of HSP90 alleviated various diseases by regulating PCD.

Inhibitors	PCD	Mechanism	Disease	Reference
DHQ3	necroptosis	Activating the cascade of RIP1-RIP3-MLKL to inducing necroptosis.	breast cancer	[58]
FW-04-806	apoptosis	Down-regulating HSP90 clients (HER2, Akt, RAF-1) and their phosphorylated forms (P-HER2, P-Akt); Inducing apoptosis.	breast cancer	[36]
C0818	apoptosis	Degrading the clients of HSP90; Inducing caspase-dependent apoptosis.	liver cancer	[144]
NVPAUG922	apoptosis	Increasing the expression of LC3II; Inducing autophagy flux.	pancreatic cancer	[145]
SNX-2112	apoptosis, autophagy	Down-regulating Bcl-2 and Bcl-XL, up-regulating Bid, cleavage- caspase-9, caspase-7, caspase-3 and PARP, and activating Caspase-8; Activating mitochondria mediated and death receptor-mediated apoptosis pathways.	melanoma	[115]
Gedunin	apoptosis	Adding sensitivity of A549 cells to apoptosis; Inhibitng the interaction between HSP90/Beclin-1/Bcl-2, leading to the down-regulation of autophagy (Beclin-1, ATG5-12 complex, and LC3) and anti-apoptotic protein Bcl-2.	lung cancer	[19]
17-AAG	apoptosis	Down-regulating c-FLIPL to promoting apoptosis.	lung cancer	[39]
CUDC-305	apoptosis	Inducing the degradation of receptor tyrosine kinases and downstream signaling molecules in the PI3K/AKT and RAF/MEK/ERK pathways.	lung cancer	[25]
Ganetespib	apoptosis	Target autophagy by destabilizing ATG7; Mediating autophagy by destabilizing ATG7.	non-small cell lung cancer	[112]
FS-108	apoptosis	Affecting the drug resistance related proteins and their downstream Akt and Erk.	non-small cell lung cancer	[60]
17-AAG	apoptosis	Inhibiting the NF-κB pathway.	chronic lymphocytic leukemia	[146]
17-DMAG	apoptosis	Inhibiting mTOR pathway; Promoting the transformation from LC3 I to LC3 II.	myeloma	[108]
shHSP90	apoptosis	Inhibiting the AKT/GSK3β/β-catenin signaling pathway and drug resistance of cancer cells.	ovarian carcinoma	[47]
Radicicol	apoptosis	Activating the caspase-8 and bid-dependent pathways and mitochondria-mediated apoptosis.	ovarian carcinoma	[98]
Curcumin	apoptosis	Inhibiting the toxic effect of MPP on SH-SY5Y cells; Reducing apoptosis.	parkinson’s disease	[23]
17-AAG	necroptosis	Decreasing the activation of RIP1/RIP3/MLKL.	heat failure	[118]
GA	necroptosis	Decreasing the Hsp90 expression to promoting instability of RIP1.	ischemic injury	[40]
HSP90i	apoptosis	Reducing infarct size; Reducing Bax/Bcl-2 ratio.	ex vivo heart perfusion system	[147]
GA	apoptosis	Decreasing the expression of MLK3; Down-regulating the activation of JNK.	ischemic injury	[83]
17AAG	apoptosis	Inhibiting IRE-1 to promoting the expression of HAX-1.	ischemic injury	[79]
GA	apoptosis	Decreasing infarct size, the expression of TNF-α, and apoptosis.	ischemic injury	[80]
GA	apoptosis	Decreasing the expression and activation of MLK3 and FasL;	ischemic injury	[48]
siHSP90	apoptosis	Up-regulating Bcl-1 and down-regulating caspase-3 and Bax.	ischemic injury	[81]
17AAG	necroptosis	Blocking the interaction of RIP1 and RIP3; Reducing the phosphorylation of RIP3.	acute respiratory distress syndrome	[120]

HSP90 inhibitors acted on the degradation and activation of clients related to PCD, then promoting the treatment of disease.

#### HSP90 in cancer

Compared with normal tissue, cancer cell shows a higher expression of HSP90 on mRNA and protein level [49]. It was reported that the concentration of HSP90 in plasma is associated with the malignant degree of the tumor, which is supported by the clinical data of more than 4000 patients with breast cancer [50]. Some clients may mutate or overexpress in pathological cells, which is alleviated by HSP90 inhibitors, indicating a therapeutic role in cancer. The cancer-related clients, including mitogen-activated protein kinase (MAPK) kinase, extracellular signal-regulated kinase (Erk) 1/2, and Akt [49, 51], take part in the proliferation, invasion and resistance of cancer cells [49, 52, 53]. For example, A synthetic inhibitor PF-4942847 exerts intensive effects on the viability of cancer cells by inducing apoptosis, delaying OS tumor growth and reducing lung metastasis [54]. Some recent studies shows that a higher level of HSP90 is found in plasma membrane and extracellular space of cancer cells, which participate in the invasion of tumor [52, 55]. For instance, the up-regulation of HSP90α enhances the aggressiveness of cancer cells by interacting with matrix metalloproteinase (MMP) 2 [56].

As antitumor drugs, triggering PCD is one of the most essential events, and the HSP90 inhibitors affect multiple PCD in various cancer have been verified [34]. Classical inhibitor GA has been reported that can induce autophagy and apoptosis in osteosarcoma cells by inhibiting the Akt signaling pathway [57]. Inhibitor 17-DMAG regulates the phosphorylation of Akt and Bcl-xl to increase apoptosis by targeting HSP90 whether in human or mouse lung cancer cells [49]. In addition, necroptosis is also an optional pathway for HSP90 inhibitor, which is related to the RIP1/RIP3/ mixed lineage kinase domain-like protein (MLKL) cascade [58].

Furthermore, the expressions of some resistance-related proteins like breast cancer resistance protein and survivin are regulated by HSP90 [47]. For example, multiple myeloma is a type of cancer with high recurrence and resistance, which is alleviated by blocking the interaction of HSP90 and CDC37 [59]. Due to the compatibility of unstable kinases with HSP90, inhibitors are beneficial to eliminate resistance after kinase inhibition in the treatment of cancer [60, 61]. 17-AAG affects radiosensitivity which is a pivotal point of clinical treatment by increasing the F-box protein 6 mediated polyubiquitination of CD147 [59]. HSP90 also is a key mediator of IFN-γ-induced adaptive immune resistance by regulating the expression of immune checkpoints like programmed death ligand 1, which exerts physiologic and pathologic effects in autoimmunity and immune escape [53]. In addition, some classical signaling pathways in cancer are affected by HSP90, such as Akt and NF-κB pathway [51, 62, 63]. In general, HSP90 inhibitors have been already demonstrated that against several cancers effectively due to its regulatory of key proteins in the development of cancer.

#### HSP90 in neurodegenerative diseases

Protein homeostasis is one of the important factors of neuronal state, and its disorder may be the main reason of some conformational diseases. It was reported that the misfolding and aggregation of symbolic proteins are partly related to HSP90 chaperone machines [64]. The characteristic Tau tangles and β-amyloid deposition are co-locating with HSP90 in Alzheimer’s disease patients, whose aggregation and degradation are regulated by HSP90 [65]. According to the study of Chen et al., the neurotoxicity caused by β-amyloid could be reduced by the HSP90 inhibitor, further promoting the normalization of synaptic function [66]. In addition, the complex consisting of HSP90, p23 and PHD2 shows a significant increase both in *vitro* and *vivo* PD models, and the clinical symptomatic relief of PD can be obtained when the assembly of complex is inhibited [67]. And the HSP90/FK506-binding protein (FKBP) 51 machine is demonstrated that involves in the activity of GR, which is regarded as a regulatory of psychiatric diseases like depression [68, 69]. In general, HSP90 chaperone machine is widely participating in the development and treatment of a variety of neurodegenerative diseases by regulating the balance between HSP90 and different co-chaperones.

Tau participates in the assembly and stabilization of microtubules and performed hyperphosphorylation and aggregation in neurodegenerative diseases [70]. HSP90 plays an essential role in the process of folding, degradation and aggregation of tau, and the relevant co-chaperones include FKBP51 and CDC37 [35, 65]. Oligomerization of Tau is synergistically triggered by the HSP90/FKBP51 machine, which is conductive to the accumulation of the toxic Tau [71]. The co-chaperone Aha1 also increases the aggregation and toxicity of Tau [72]. In contrast, protein phosphatase 5 and cyclophilin 40 promote phosphorylation and decomposition of the aggregating Tau [65].

Similarly, TAR DNA binding protein (TDP) 43 is also a client of HSP90, whose aberrant aggregation is a signature of amyotrophic lateral sclerosis [73]. As the study of Lin et al., the toxicity of TDP-43 is regulated by the specific interaction between HSP90/stress-inducible phosphoprotein (Sti) 1 machine and TDP-43 [74, 75]. The appropriate expression of Sti1 reduces the toxicity of TDP-43, while the abnormal Sti1 promotes the dysfunction of the neuron. In addition, HSP90/CDC37 is associated with the nuclear location of TDP-43 [76]. The all above suggest that the HSP90 machine plays a significant effect on neurodegenerative diseases.

#### HSP90 in cerebro-cardiovascular diseases

The function of endothelial nitric oxide synthase, endothelial growth factor receptor and other vascular-related proteins are regulated by HSP90, which is related to the circulatory system [77]. It was reported that the Erk and Heme-Oxygenase-1 signaling are activated by suppressing the interaction of CDC37/HSP90, which could effectively reduce the infarct area, fibrosis and macrophage infiltration in the myocardial ischemia/perfusion model [78]. And inhibiting apoptosis is also the mechanism by which HSP90 inhibitors exert their protective effect [79]. GA reduces apoptosis in the process of myocardial injury by mediating the complement system and JNK signaling pathway [80].

HSP90 is also involved in cerebrovascular disease. Numerous studies demonstrated that HSP90 is significantly increasing in the model of cerebral ischemia-reperfusion injury [81]. The HSP90 inhibitor GA shows obvious neuronal protection both in the whole and focal cerebral I/R model, owing to the up-regulation of HSP70 and HSP25 in neurons [82]. In the model of four-vessel occlusion ischemic on rat, the intensive association of HSP90 and MLK3 is reversed by GA, which exerts a strong neuroprotection [83]. In addition to necroptosis, the inhibition of HSP90 is also related to apoptosis, autophagy and other PCD in stroke [48]. The another therapeutic mechanism of HSP90 inhibitor for cerebrovascular disease is maintaining the function of blood-brain barrier (BBB), which easily affects by inflammation under hypoxia condition [84]. In the study of Zhang et al., injecting siHSP90 alleviates oxidative stress and inflammation in the model of I/R[81]. And 17-DMAG is considered to play a protective role by down-regulating MMP9 to maintain BBB [85].

#### HSP90 in other diseases

Some studies have been demonstrated that HSP90 is involved in pulmonary fibrosis, and 17-AAG could decrease fibrosis and MMP activity to alleviate idiopathic pulmonary fibrosis [86]. In addition, HSP90 is associated with the activation of glial cells in the spinal cord that are involved in the typical pain signaling cascade events [87]. Treating with 17-DMAG could relieve abnormal pain induced by exercise and monoarthritis, which is related to inflammatory cascade [88, 89]. In the case of low oxygen, HSP90α combines with the low-density lipoprotein receptor-related protein 1 cytoplasm tail to stabilize the receptor on the cell surface, and the Hsp90β is secreted into the cell space to increase cell movement, to promote wound healing by interacting with the low-density lipoprotein-related protein-1 receptor signaling [90].

Overall, the treatment strategy of targeting HSP90 has been widely studied, but its specific role in diseases remains not fully clarified. HSP90 is generally thought to be up-regulated in response to stress rather than cause disease. However, the up-regulation of HSP90 is beneficial to the survival of cancer cells and the mutation of cancer-related proteins, which is the reason for the use of inhibitors in cancer treatment. Furthermore, HSP90 in cancer cells is distinguished from the ordinary cells by the greater activities, extracellular localization and special post-translational modifications [45]. There are more than 30 different post-translational modifications of HSP90 in cells, and the change of modification induced by exogenous stimulus might be one of the mechanisms of its negative role in diseases. For example, alcohol induces the acetylation of HSP90 to decrease the interaction with eNOS, further leading to liver injury [91]. And the S-nitrification of HSP90 is observed in atherosis [30]. And for neurodegenerative diseases like AD, the imbalance of the chaperone system including HSP90 might be an important factor in its pathogenesis. In general, HSP90 has a unique mechanism under the disease state, but its specific mechanism still needs to be supplemented.

## The relationship of HSP90 and multiple PCD

**Fig. 3:**
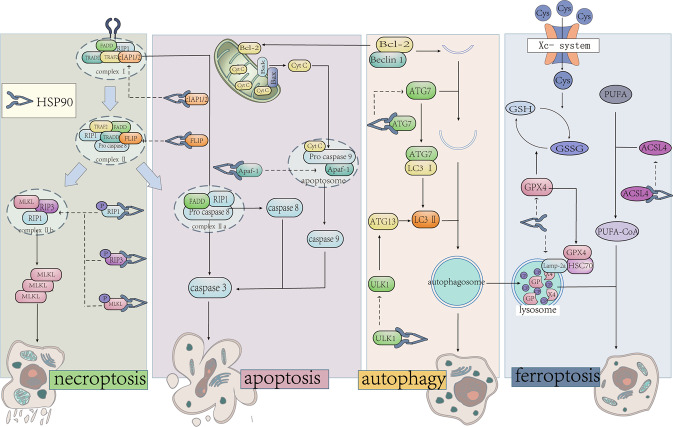
HSP90 mediates multiple PCD. HSP90 plays an important role in the PCD regulation network. Forming complex with co-chaperone and clients to regulate their phosphorylation and stability is the main mechanism. And the clients of HSP90 are distributed in various processes, including the RIP1, RIP3 and MLKL in necroptosis, Bcl-2 and cellular-FLICE inhibitory protein (FLIP) in apoptosis, ULK1 and ATG7 in autophagy, GPX4 and ACLS4 in necroptosis.

As a fundamental process of cells, there are complex connections among multiple PCD, and HSP90 is one of the common regulatory nodal in apoptosis, autophagy, necroptosis, ferroptosis and other PCD. (Fig. 3).

### HSP90 and apoptosis

Apoptosis is the earliest cognitive PCD, which occurs ubiquitously in various diseases by eliminating aberrant cells through intrinsic and extrinsic pathways [92]. The intrinsic pathway is caused by internal stress signals like DNA damage, resulting in mitochondrial outer membrane permeabilization(MOMP) [93]. Then, the Cyt C released from mitochondria assembles the apoptotic bodies to facilitate the activation of the caspase cascade and further induce apoptosis ultimately [94]. And the extrinsic pathway can promote the assembly of the DISC to induce the maturation of caspase8 and triggers the caspase cascade to mediate apoptosis [95]. With further study of the mechanism, both the intrinsic and extrinsic pathways are believed to be regulated by HSP90.

HSP90 is closely related to multiple processes of intrinsic apoptosis. For example, HSP90 decreases the release of Cyt C by interacting with Bcl-2 [96]. And the G-TPP could cause MOMP and the release of Cyt C by inhibiting TRAP-1 [97]. HSP90 also takes part in the assembly of apoptotic bodies by regulating apoptotic protease activating factor 1 and is associated with the cleavage and function of numerous caspases, including caspase3, 6, 9, and so on [98–101]. Some N-terminal inhibitors also could regulate apoptosis by activating HSF-1, which is beneficial for the up-regulation of HSP70 and HSP27 [102].

In the extrinsic pathway, c-FLIP is an essential negative regulatory which blocks the activation of caspase 8/10 in DISC [103]. 17-AAG induces apoptosis in lung cancer cells by decreasing the expression of c-FLIP, suggesting that HSP90 may mediate apoptosis by impacting the degradation of c-FLIP [39, 104, 105]. In addition, the MG132, a proteasome inhibitor, inhibits the down-regulation of c-FLIP in CALu-1 cells after 17-AAG treatment [39], which proves the regulation of 17-AAG in apoptosis owes to mediating the degradation of c-FLIP via the proteasome pathway.

### HSP90 and autophagy

Autophagy is defined as a process of removing damaged proteins and organelles by engulfing them into vesicles to form autophagosomes [106], including microautophagy, chaperone-mediated autophagy (CMA), and macroautophagy. CMA is described as a process that degrades specific clients by transporting them into lysosomes when recognized by lysosome-associated membrane protein type (LAMP) 2a [107]. And the stability of LAMP-2a is regulated by HSP90 [108]. Generally, autophagy is closely related to the degradation of clients which includes lysosome and ubiquitinated protease pathways. For example, IKK is selectively degraded when HSP90 is inhibited, and the inhibition of ATG5 can reverse IKK degradation [38], suggesting that HSP90 may lead to the degradation of IKK through the lysosomal pathway. In contrast, the protease inhibitor MG132 interrupts the degradation of RIP3, which is related to the ubiquitinated protease pathway [41].

In addition, HSP90 is associated with several key proteins in macroautophagy, such as ULK1, Beclin-1, ATG7, and so on [109]. ULK1, a mammalian homolog of ATG1, is involved in the nucleation and extension process of the autophagosome, whose function is maintained by forming a complex with HSP90 and CDC37 on its N-terminal kinase domain [110]. On one hand, the interaction between HSP90 and ULK1 contributes to the autophosphorylation of ULK1 at Ser1047, which is interrupted by the treatment of HSP90 inhibitor [110]. On the other hand, 17-AAG does not affect the mRNA level of ULK1, but it could decrease the homeostasis of ULK1, indicating that the interaction between HSP90 and ULK1 is in favor of the stabilization of ULK1. In addition, ATG13 is a substrate of ULK1 and could be phosphorylated by ULK1 at Ser318, whose phosphorylation needs HSP90/CDC37/ULK1 complex [110, 111]. HSP90 is also related to the localization of ATG13, which is described as translocating to damaged mitochondria to mediate its elimination [112].

Beclin-1 is a key regulatory in early autophagy whose function depends on HSP90 [113]. HSP90 forms a complex with Beclin-1 through an evolutionarily conserved domain to maintaining its stability and phosphorylation [69, 114]. GA separates Beclin-1 from HSP90, further promoting its degradation through the ubiquitinated protease pathway [114]. In addition, SNX-2112 could inhibit the formation of the ATG7/caspase9 complex, which is a key to the alteration of apoptosis and autophagy [115]. GA also affects the interaction between HSP90 and ATG7 by disrupting the stability of ATG7 [112]. It was also reported that the regulation of HSP90 inhibitor on autophagy is related to the rate of LC3II/I and the formation of autophagosomes [116, 117].

### HSP90 and necroptosis

Compared to necrosis that had been considered as unregulated, increasing evidence indicates that there is a caspase-independent PCD defined as necroptosis, characterized by the loss of cell membrane integrity and release of cytoplasmic contents [3]. Necroptosis is strictly regulated by the RIP1/RIP3/MLKL pathway, and the HSP90 inhibitor involves in the stability, phosphorylation, and expression levels of RIP1, RIP3 and MLKL in necroptosis [17, 40]. For example, the higher expression and hyperphosphorylation of RIP1, RIP3 and MLKL in the model of heart failure would be reversed by HSP90 inhibitor [118]. The expression of RIP1 and RIP3 is inhibited by 17-AAG which could be reversed by CDC37 knockdown, suggesting that activation of RIP3 is related to the HSP90/CDC37 complex [119]. In addition to CDC37, p23 is also co-located with RIP3, affecting its phosphorylation [120]. The correlation between HSP90 and RIP3 expression remains controversial. Although most studies have shown that HSP90 inhibitors simultaneously reduce the phosphorylation of RIP3 in disease models [40], 17-AAG has no effect on the abundance of RIP3 and merely regulates its function [119]. The duration of HSP90 inhibitors effecting on cells is also critical to the function of RIP3, which demonstrated that short-term inhibition of HSP90 may lead to conformational changes of RIP3, and the degradation of RIP3 via ubiquitinated protease pathway is mediated by long-term inhibition of HSP90 [41, 121, 122].

Activity of HSP90 is also essential for the processes of phosphorylation, oligomerization and membrane translocation of MLKL [123, 124]. Previous studies show that MLKL is phosphorylated at Ser227 and Ser358 during necroptosis, which is strongly inhibited by 17-AAG [122]. HSP90 is also vital to promote the oligomerization and translocation of MLKL, though the interaction with HSP90 is weak or transitory [122–124]. Considering that MLKL is downstream of this cascade, the RIP3-deficient fibroblast cell is used for determining that HSP90 inhibitors can directly regulate MLKL to mediate necroptosis [122]. In general, HSP90 takes part in multiple processes of necroptosis regulated by different HSP90 inhibitors.

### HSP90 and ferroptosis

Ferroptosis is an emerging iron-dependent cell death way, which is characterized by membrane rupture and vesiculation, mitochondrial atrophy, decrease of the mitochondrial ridge, and an increase in membrane density [2]. Some studies show that HSP90 is a potential target for ferroptosis, while its role in ferroptosis remains controversial [18, 125]. Su et al. found that HSP90 inhibitor GA promotes the depletion of GSH to accelerates the occurrence of ferroptosis [125]. On the contrary, another HSP90 inhibitor CDDO is beneficial in reducing ferroptosis [18]. According to the study of Wu et al., CDDO significantly inhibits ferroptosis by affecting the expression of GPX4, which is one of the most classical biomarkers of ferroptosis [18, 126].

A previous study shows that GPX4 is the substrate of CMA, which is affected by LAMP-2a [108, 127]. The overexpressed LAMP-2a promotes CMA to decrease the expression of GPX4, which enhances the sensitivity to erastin-induced ferroptosis, and an obvious high expression of GPX4 is detected in LAMP-2a knockdown cells [128]. The interaction of HSP90 and Lamp-2a shows a significant increasing when the cells treat with erastin [18, 108]. Another latest study also demonstrates that HSP90 plays a positive role in ferroptosis by regulating ACSL4. As a key biomarker of ferroptosis, ACSL4 involves in lipid peroxidation, whose expression is related to the interaction of HSP90 and Drp1 [129].

### HSP90 and other PCD (PANoptosis)

In addition to the above, some studies have shown that HSP90 is also associated with other PCD, such as pyroptosis. Pyroptosis is a recently discovered PCD accompanied by an inflammatory response, which is characterized by rapid plasma membrane rupture, DNA damage, and the release of pro-inflammatory cytokine [130]. And the NLRP3/caspase-1/GSDMD pathway is considered to be the key to regulating pyroptosis. Inhibition of NLRP3 inflammasome is a reliable therapeutic target for a variety of inflammatory diseases, and the interaction of HSP90 and NLRP3 is related to its stability and activation, further regulates downstream IL-1β secretion and pyroptosis[131]. Normally, NLRP3 is inactivated when it binds to HSP90, and upon receiving an inflammatory signal, the interaction is blocking to prompting the activation of NLRP3 and the initiation of subsequent inflammatory cascades [132]. Finally, the complex correlations between HSP90 and emerging PCD, like Cuproptosis, NETosis and PANoptosis, remain to be further investigated.

## Crosstalk between HSP90 and multiple PCD

In the studies of targeting for HSP90, some researchers have found that HSP90 may involve in the selection of multiple PCD. As reported, the inhibition of HSP90 could transform necroptosis induced by DD receptor into apoptosis [133]. And different HSP90 inhibitors selectively activate or inhibit multiple PCD, even in the same objects. For example, DHQ3 induces necroptosis by activating the RIP1/RIP3/MLKL pathway in human breast cancer cells, while 17-DR induces caspase-3 and caspase-8-dependent apoptosis [58]. In addition, HSP90 inhibitors also appear under different regulations when affecting multiple PCD simultaneously (Table 2). According to the research of Yan et al., HSP90 is a critical regulator of necroptosis and apoptosis, whose inhibitor alleviates necroptosis and promote the activation of apoptosis [97]. In another study, the inhibition of HSP90 could reduce both necroptosis and apoptosis in nucleus pulposus-derived stem cells [17]. The specific regulation is both related to types of inhibitors and pathological context of cell. For, example, 17-AAG, a widely used inhibitor, induces apoptosis in lung cancer cells but reduces apoptosis in a rat CCI model [39].

**Table 2:** HSP90 inhibitors regulated multiple PCD simultaneously.

HSP90 inhibitors	PCD	Function	Cells	Reference
gedunin	autophagy	reduce	Lung cancer cells	[19]
	apoptosis	induce		
GA	autophagy	induce	Osteosarcoma cells	[57]
	apoptosis	induce		
GA	autophagy	induce	kidney cells	[148]
	apoptosis	reduce		
SNX-2112	autophagy	induce	Melanoma cells	[115]
	apoptosis	induce		
17-AAG	autophagy	induce	Hepatocellular carcinoma	[149]
	apoptosis	induce		
17-DMAG	autophagy	reduce	B-ALL cells	[150]
	apoptosis	induce		
G-TPP	autophagy	induce	Hep3B cells	[97]
	apoptosis	induce		
	necroptosis	induce		
BIIB021	apoptosis	reduce	NPSCs	[17]
	necroptosis	reduce		
CDDO	necroptosis	reduce	HT-22	[18]
	ferroptosis	reduce		

The inhibitors regulated different PCD in the same objects. And the different inhibitors mediated the same PCD in a similar or contrast way.

**Fig. 4:**
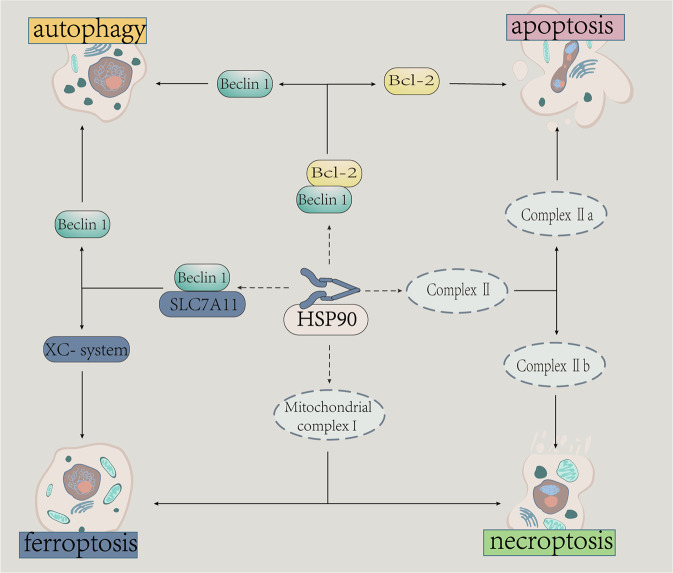
HSP90 relates to the selection of cell death forms in cells. There are complex connects among multiple PCD, and the relation of them are not fixed but flexible which depends on the physiological and pathological conditions. HSP90 regulates some key procedures of the PCD transformation, including the Beclin1/Bcl-2 complex, Beclin1/SLC7A11 complex, mitochondrial complex I and complex II.

With the deepening of relevant researches, parts of complexes and proteins are regarded as key nodal in the complex regulatory network of PCD, selectively promoting cells towards different PCD, including complex II, Beclin1-Bcl 2, and some caspases [134].(Fig. 4). Mechanistically, necroptosis and extrinsic apoptosis share a common initiation, which is separated by the assemble of complex I and II. cIAP1/2 is one of the key regulatory, exerting anti-apoptotic function [135]. When cIAP1/2 is inhibited, the assembly of complex IIa is increasing to activate the caspase cascade and induce apoptosis, which is consisted of RIP1, FADD, and caspase-8 [136]. When RIP1 deubiquitinated and caspase-8 inhibited, the assembly of complex IIb induces necroptosis promoted after the binding of RIP3 and RIP1. Inhibitor CDDO could inhibit necroptosis by disrupting the formation of complex IIb [18]. Another determining element is the balance of c-FLIP and RIP1/RIP3/MLKL whose expression and function are regulated by HSP90 [39].

Beclin-1/Bcl-2 is also an important common regulatory nodal between apoptosis and autophagy, whose stability requires the involvement of HSP90 [137]. Gedunin targets HSP90 to mediate the interaction of Beclin-1/Bcl-2 and endoplasmic reticulum stress, then regulates the transformation between apoptosis and autophagy [19]. Beclin1 could also disrupt the Xc^-^ system by interacting with SLC7A11, which may mediate the correlation between autophagy and ferroptosis [138]. In addition, mitochondrial complex I is an important nodal for autophagy, necroptosis and ferroptosis, which could be inhibited by celastrol [139, 140]. In addition to apoptotic activity, some caspases can also participate in other physiological activities [141]. For example, caspase 9 is involved in the initial autophagosome formation when its apoptotic activity is inhibited by interacting with ATG7, which could be regulated by an HSP90 inhibitor [6, 112, 142].

## Conclusions and perspectives

Since PCD is closely related to the process of disease development, drugs usually take effect by eliminating aberrant cells and protecting normal cells. The HSP90 inhibitors have been used in the research of diseases due to the excellent effect in this respect. However, there are still many problems need to be solved in this process. For example, some N-terminal inhibitors regulate PCD by binding to the NTD of HSP90, which would up-regulate the HSP70-related cytoprotection to cause unsatisfactory results [33]. This phenomenon promotes the development of CTD and MD inhibitors which would not induce this protection. Furthermore, although HSP90 inhibitors have shown promising therapeutic effects in related mechanistic studies, they have not performed as expected in clinical trials. The dissatisfactory specificity of inhibitor is one of the main limiting factors, which is caused by the sequence identity of the four isoforms, especially the 85% similarity between HSP90α and HSP90β [8]. So, the researches on isoform-selective inhibitors are still continually deepened, which is conductive to reducing the pan-inhibition of Hsp90. In the study of Chaudhury et al., the reported complex shows the greatest selectivity towards HSP90β, and even more than 370 fold compared with HSP90α [143]. In general, although the problems of targeting HSP90 in disease treatment have revealed, it still be a feasible approach because these problems could be gradually solved.

Overall, according to relevant studies of targeting HSP90 for diseases therapy, the feasibility and prospects are as follows: [1] Due to the complex interactions of HSP90, co-chaperone and clients, HSP90 inhibitors are designed to regulating key proteins in diseases by blocking the combination. However, the current studies mainly focus on single PCD, and the effect of inhibitors on various PCD is complex. How to select the appropriate inhibitors to promote cell survival or inhibit cell activity as a whole may be one of the important problems for the situation of HSP90 as a therapeutic target. [2] As far as cancer treatment is concerned, the mechanism of targeting HSP90 to regulate apoptosis and necroptosis has already presented lots of relevant studies, but the relationship between HSP90 and some emerging cell death pathways should be further explored, including ferroptosis, pyroptosis, and cuproptosis. [3] N-terminal inhibitors of HSP90 can activate the heat shock response and increase the expression of HSP70, which exerts strong cellular protection. The low-dose inhibitors may acutely activate the heat shock response to alleviate disease without extensive cytotoxicity, so HSP90 inhibitor with an appropriate dose may be a vital event in disease treatment. In general, the role of HSP90 in disease and regulation in multiple PCD still has great prospects.

## Data Availability

Original data used for this report (although not applicable) will be made available upon request to the corresponding author.
